# The Effect of Fermentable, Oligosaccharides, Disaccharides, Monosaccharides, and Polyols (FODMAP) Meals on Transient Lower Esophageal Relaxations (TLESR) in Gastroesophageal Reflux Disease (GERD) Patients with Overlapping Irritable Bowel Syndrome (IBS)

**DOI:** 10.3390/nu14091755

**Published:** 2022-04-22

**Authors:** Suppawatsa Plaidum, Tanisa Patcharatrakul, Wachinee Promjampa, Sutep Gonlachanvit

**Affiliations:** 1 Division of Gastroenterology, Department of Medicine, King Chulalongkorn Memorial Hospital, The Thai Red Cross Society, Bangkok 10330, Thailand; suppawatsap@gmail.com (S.P.); dr_tanisa@yahoo.com (T.P.); wachinee.promjampa@gmail.com (W.P.); 2 Center of Excellence on Neurogastroenterology and Motility, Faculty of Medicine, Chulalongkorn University, Bangkok 10330, Thailand

**Keywords:** non-constipation irritable bowel syndrome, GERD, wheat, rice, low FODMAPs, high FODMAPs, intestinal gas, hydrogen, gastrointestinal symptoms

## Abstract

A randomized crossover study in eight patients (6 F, age 57 ± 13) with overlapping GERD-IBS (non-constipation) was conducted to evaluate the effects of rice noodle vs. wheat noodle meals for breakfast and lunch on postprandial TLESR, intestinal gas production, and GERD/GI symptoms. Results: Wheat ingestion was significantly associated with more frequent TLESR after lunch than rice (5.0 ± 0.7 vs. 1.9 ± 0.3 times/2 h, *p* = 0.01). After lunch, wheat ingestion was significantly associated with higher H_2_ and CH_4_ levels compared to rice ingestion (*p* < 0.05), while H_2_ and CH_4_ levels before lunch were similar (*p* > 0.05). The area under curve of H_2_ concentration until 2 h after lunch significantly correlated with the TLESR number (*r* = 0.69, *p* = 0.04). Postprandial regurgitation (2.9 ± 1.2 vs. 0.4 ± 0.2), bloating (7.0 ± 0.4 vs. 3.1 ± 0.9), satiety (7.7 ± 0.4 vs. 3.5 ± 0.9), and belching (3.8 ± 1.2 vs. 1.1 ± 0.6) symptom scores were significantly greater after wheat compared to rice noodle ingestion (*p* < 0.05). Conclusion: Wheat noodle meals, part of a high FODMAP diet, induced a higher frequency of TLESRs, a higher GERD, and higher upper-GI symptom scores than rice noodle meals, part of a low FODMAP diet, in patients with overlapping IBS-GERD. These effects were associated with more intestinal gas production. Thus, a low FODMAP diet may relieve GERD symptoms in GERD patients with overlapping IBS.

## 1. Introduction

Gastroesophageal reflux disease (GERD) is a common gastrointestinal disorder that affects the worldwide population. A systematic review and meta-analysis have demonstrated that the prevalence of gastroesophageal reflux (GER) symptoms in individuals with irritable bowel syndrome (IBS) is four-fold than in the general population, and an overlap between the two conditions has been reported for up to one-third of IBS patients [1]. Likewise, the prevalence of IBS in GERD patients ranges from 32% to 47% [2]. A previous study showed that overlapping GERD and IBS was associated with a lower GERD response to medical treatment and a lower quality of life than a single functional gastrointestinal disorder (FGID) [3]. 

A low fermentable, oligo-, di-, and monosaccharides, and polyols (FODMAP) diet plays an important role in IBS treatment. Randomized controlled studies reported the benefit of consuming a low FODMAP diet on gastrointestinal symptoms and quality of life in patients with IBS [4,5,6,7,8,9,10,11,12,13,14,15,16]. In contrast, the role of a low FODMAP diet in GERD has not been well understood. Although lifestyle modifications, including avoiding a list of foods such as alcohol, caffeine, and fatty food, are recommended as a non-pharmacological treatment of GERD [17], modifications of food ingestion have demonstrated a limited benefit [18], and the role of foods on the pathogenesis and treatment of GERD has been uncertain for decades [18]. Most studies have focused on energy and lipids and the results do not indicate that food plays a significant role in GER symptoms [19,20]. Carbohydrates have not been clearly investigated. Up to 20% of carbohydrates escape digestion and absorption in the human small intestine, and they are mostly fermented into short-chain fatty acids (SCFAs) and hydrogen by the colonic microflora. Studies showed that the exposure of the proximal colon to SCFAs contributes to the regulation of gastric motility [21], and lower esophageal sphincter (LES) function [22]. A colonic fermentation of ingested lactulose and direct colonic infusion of a mixture of SCFAs, resulting in a relaxation of the proximal stomach [21], then triggered transient lower esophageal sphincter relaxations (TLESRs) [23,24]. Thus, a high FODMAP diet may aggravate gastroesophageal refluxes and GERD symptoms. However, the effects of FODMAPs on lower esophageal sphincter functions, GER, and GERD symptoms are incompletely understood or have not been well explored. 

Therefore, this study aimed to evaluate the effects of low and high FODMAP meals on TLESR, intestinal gas production, postprandial GER, typical GERD, and other gastrointestinal (GI) symptoms in patients with overlapping GERD-IBS.

## 2. Materials and Methods

### 2.1. Study Subjects

Participants aged 18–65 years old, who had typical GER (bothersome heartburn and/or regurgitation) symptoms and non-constipation type IBS according to the Rome III criteria, were recruited from the out-patients clinic at the Center of Excellence on Neurogastroenterology and Motility, Faculty of Medicine, Chulalongkorn University, Bangkok, Thailand. The exclusion criteria were a previous history of abdominal surgery, pregnancy, major psychological disorders, a history of allergy to the test meals, and comorbid pulmonary conditions such as chronic obstructive pulmonary disease (COPD) and asthma. All subjects did not use acid suppressants, probiotic supplements, prokinetics, laxatives, antibiotics, over-the-counter drugs, or medications that affect GI functions and symptoms during a 2-week run-in and the study period; they also recorded their food diary for three days before the study. The patients with constipation-predominant IBS, defined by the Rome III criteria, were excluded from this study using a symptom questionnaire with the Bristol Stool Form Scale (BSFS). 

### 2.2. Study Design

After a 2-week run-in, patients who still had an overall GI symptom severity of more than 5 out of 10 cm of the visual analog scale (VAS) during the past week were enrolled. All subjects were randomly assigned using a sealed and opaque envelop that a study coordinator opened before the study day to determine the two different test meal allocation (rice noodles or wheat noodles), before a crossover. A one-week washout period was judged sufficient time for the meal in the previous period to have washed out, as the participants had stool frequency at least 3 times per week. After overnight fasting, all subjects reported at the Center of Excellence on Neurogastroenterology and Motility, Chulalongkorn University, at 7.30 AM, when the baseline GI symptom scores and BSFS were assessed. All subjects ingested a standard meal of 250 g rice noodles or 250 g wheat noodles at 8.00 AM (breakfast) and 12.00 PM (lunch). Although the rice and wheat noodles looked different, all patients were not informed about the major component of the noodles, and the term “FODMAPs” was not mentioned in the patient’s information sheet. Exhaled breath hydrogen (H_2_) and methane (CH_4_) gas were acquired from all subjects at fasting and after breakfast every 15 min for 8 h. A high-resolution esophageal impedance manometry (HRiM) catheter (outer diameter of 4.2 mm and 36 circumferential sensors separated by 1 cm intervals with 18 pairs of impedance sensors, Medtronic Inc., Shoreview, MN, USA) was placed nasally at 5 min before lunch for the monitoring of the TLESR events for a 2-h postprandial period. The recording was performed in a fully upright position, and the catheter was fixed in place with at least three intragastric sensors. Although the rice and wheat noodles looked different, all patients were not informed about the FODMAP component in each meal. The investigators who evaluated TLESR events and symptoms and measured the breath H_2_ and CH_4_ gas levels were blinded to the test meals. The GI symptoms, including typical GERD symptoms (heartburn, regurgitation), bloating, satiety, abdominal pain, stool urgency, nausea/vomiting, belching, chest pain/discomfort, and flatulence were evaluated every 15 min during 2-h after lunch using 10 cm VAS, where 0 indicated no symptoms and 10 represented the worst symptoms. All subjects gave their informed consent before they participated in the study. The study was conducted following the Declaration of Helsinki, and the protocol was registered in the Thai clinical trial number TCTR 20210608005. It was approved by the Ethics Committee of Faculty of Medicine, Chulalongkorn University (Project identification code 678/62).

### 2.3. Interventional Meals

All study meals were made from 90 g dry weight of rice or wheat flour and cooked as 250 g cooked weight noodles, as described in our previous study [25]. No vegetable or other fermentable ingredient such as garlic or soy sauce was added to avoid intestinal gas production from other sources. In each study, participants took the test meal for breakfast and lunch. A glass of water (250 mL) was allowed with the test meals, and subjects were asked to finish their meal within 15 min. No food or drink was allowed between test meals or after lunch. We recorded the amount of food eaten in every meal. According to the U.S. department of agriculture (USDA) Food Composition Database (https://ndb.nal.usda.gov/ndb/search/list), accessed on 1 February 2022 [26], the total energy in each serving size for wheat and rice noodle in this study was 440 kcal and 450 kcal, respectively. The amount of carbohydrate, protein, fat, and fiber contents in wheat and rice noodles serving were 42, 32, 14, and 2 g and 50, 30, 14, and <1 g, respectively.

### 2.4. Breath Tests

Breath samples were collected using 250 mL sample holding bags (Quintron Instrument Co., Inc., Milwaukee, WI, USA). All breath samples were analyzed for H_2_ and CH_4_ using a Quintron Microlyzer Model DP Plus (Quintron Instrument Co., Inc., Milwaukee, WI, USA) and reported in parts per million (ppm). In the evening before the study date, all subjects were asked to control carbohydrate ingestion and avoid poorly absorbable carbohydrates, which may affect the hydrogen breath test study on the following day. Subjects were asked to maintain good oral hygiene during the breath testing phase by brushing their teeth before taking their first breath sample [27]. The baseline gas sample was a fasting sample taken before breakfast, then every 15 min for 8 h. 

### 2.5. Evaluation of TLESRs

The TLESRs were evaluated immediately after the subjects finished lunch and monitored for 2 h. A TLESR was analyzed using ManoView ESO software version 3.3 (Medtronic Inc., Shoreview, MN, USA) and defined as a spontaneous fall in LES pressure, occurring in the absence of a pharyngeal swallow signal for 4 s to 2 s after the onset of the LES relaxation. The presence of swallowing was allowed if the LES relaxation duration was longer than 10 s in the absence of multiple swallows. The duration of LES relaxation was defined as the time during which LES pressure was ≤50% of basal LES pressure within 10 s before the onset of LES relaxation. The TLESR may occur with or without a GER event, identified by a retrograde propagation of the decrease in impedance [28]. 

### 2.6. Statistical Analysis

The primary outcome was the number of TLESR after the test meal ingestion, evaluated by the HRiM system, and was compared between the test meals. Secondary outcomes were the typical GERD and GI symptom scores after lunch, and intestinal H_2_ and CH_4_ production collected from the breath samples over an 8 h study period after breakfast, which were compared between the interventional meals. The sample size was calculated based on the best data from a previous study of TLESR events influenced by colonic fermentation in symptomatic GERD patients [29]. To determine at least a 50% difference between rice and wheat with 80% power at α = 0.05, at least eight subjects were needed for this crossover study.

A comparison of TLESR numbers, GI symptoms, H_2_, and CH_4_ gas levels, between two groups were analyzed by paired *t*-test and Wilcoxon sign rank test depending on data distribution. A *p* value of less than 0.05 was defined as statistical significance. Data were expressed as mean ± SD or median (interquartile range) as appropriate. The repeated measures analysis of variance was also performed to determine whether there were period effects, sequence effects, and carryover effects that can arise in a crossover trial. The data were analyzed using SPSS software version 26.0 for Windows.

## 3. Results

Eight patients (6 females, age 57 ± 13 years) with overlapping typical GER symptoms and non-constipation type IBS were included. The patients’ BMI was 23.3 ± 2.7 kg/m^2^. The duration of GERD and IBS symptoms was 5.5 ± 1.3 and 7.8 ± 1.7 months, respectively. All patients underwent upper GI endoscopy after GERD onset and all of them had no reflux esophagitis. The median BSFS during the past month before study enrollment was 5 (range 4–6) with a stool frequency of 7 (4–9.5) times per week. Baseline typical GERD and overall symptom severity (VAS 0–10) on the study day was 2.0 (1.0–3.0) and 6.0 (5.3–7.0), respectively. All patients took the assigned wheat noodles and rice noodles at a similar amount and completed the studies without adverse events. The patients’ baseline symptom scores of each symptom were not significantly different when comparing between wheat and rice noodles (*p* > 0.05) (Table 1).

### 3.1. Effects of Wheat vs. Rice Ingestion on TLESR

**Figure 1:**
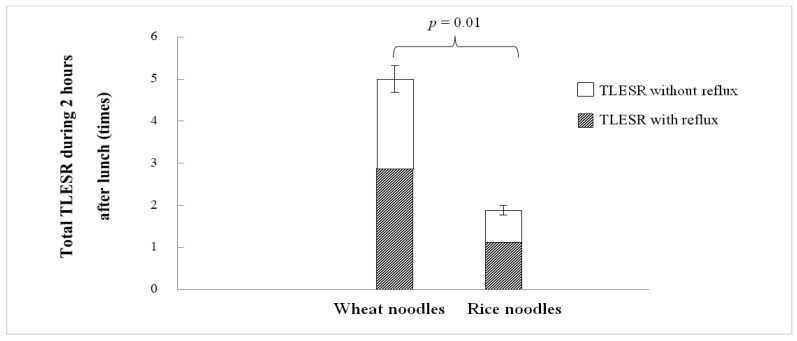
The total number of TLESR (transient lower esophageal sphincter relaxations) and TLESR associated with and without gastroesophageal reflux during 2 h after lunch comparing wheat and rice noodle ingestion.

**Figure 2:**
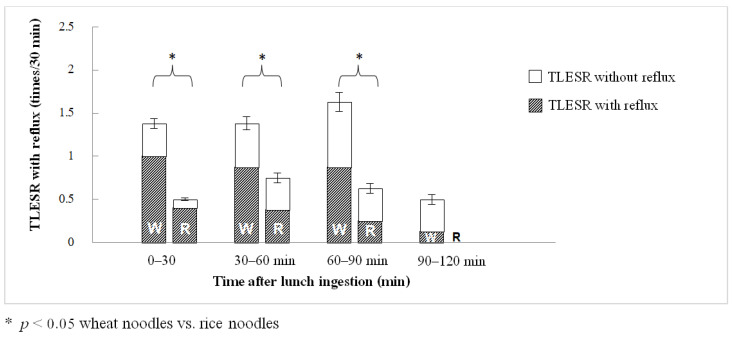
The number of TLESR per 30 min during 2 h after lunch comparing wheat noodle (W) and rice noodle (R) ingestion.

During the 2-h period after lunch, wheat noodle ingestion significantly produced more TLESR events than rice noodle ingestion (5.00 ± 0.68 vs. 1.88 ± 0.30 times/2 h, *p* = 0.01; Figure 1). The difference was statistically significant during the first 30 min of recording (1.38 ± 0.32 vs. 0.50 ± 0.19 times/30 min, *p* < 0.05), 30–60 min (1.38 ± 0.32 vs. 0.75 ± 0.16 times/30 min, *p* < 0.05), and 60–90 min postprandial (1.63 ± 0.38 vs. 0.63 ± 0.18 times/30 min, *p* < 0.05). The number of TLESRs decreased after 90 min (wheat ingestion 0.50 ± 0.27 times/30 min) with no TLESR detected in all subjects during 90–120 min after rice ingestion (Figure 2). The total number of TLESR associated with reflux after wheat noodle ingestion tended to be higher than rice noodle ingestion, but it did not reach statistical significance (2.88 ± 0.72 vs. 1.13 ± 0.13 times/2 h, *p* > 0.05; Figure 1). The number of swallows demonstrated by pharyngeal contraction during TLESR monitoring for 2 h after lunch for the rice and wheat meal was similar: 44 (28–83) times and 45 (34–82) times, respectively (*p* > 0.05). The example of esophageal manometry tracing of a patient was shown in the supplement link (Figure S1).

### 3.2. Effects of Wheat vs. Rice Ingestion on GER and Other GI Symptoms

The average symptom score of each individual during 2 h after lunch were compared between wheat and rice noodle ingestion. After wheat noodle ingestion, a regurgitation severity score was significantly higher than rice (1.5 (0.0–6.1) vs. 0.3 (0.0–0.9), *p* < 0.05). The heartburn symptom scores also tended to be more severe after wheat ingestion than rice ingestion, but it did not reach the statistical significance (2.0 (0.1–3.6) vs. 0.3 (0.0–1.6), respectively, *p* = 0.07). Moreover, wheat noodles were associated with significantly more severe other GI symptoms than rice noodles, including bloating, satiety, and belching (*p* < 0.05). Abdominal pain, stool urgency, nausea/vomiting, chest discomfort, and flatulence severity were not significantly different after a wheat and rice test meal (Table 2).

### 3.3. Effects of Wheat vs. Rice Ingestion on Intestinal Gas Production

**Figure 3:**
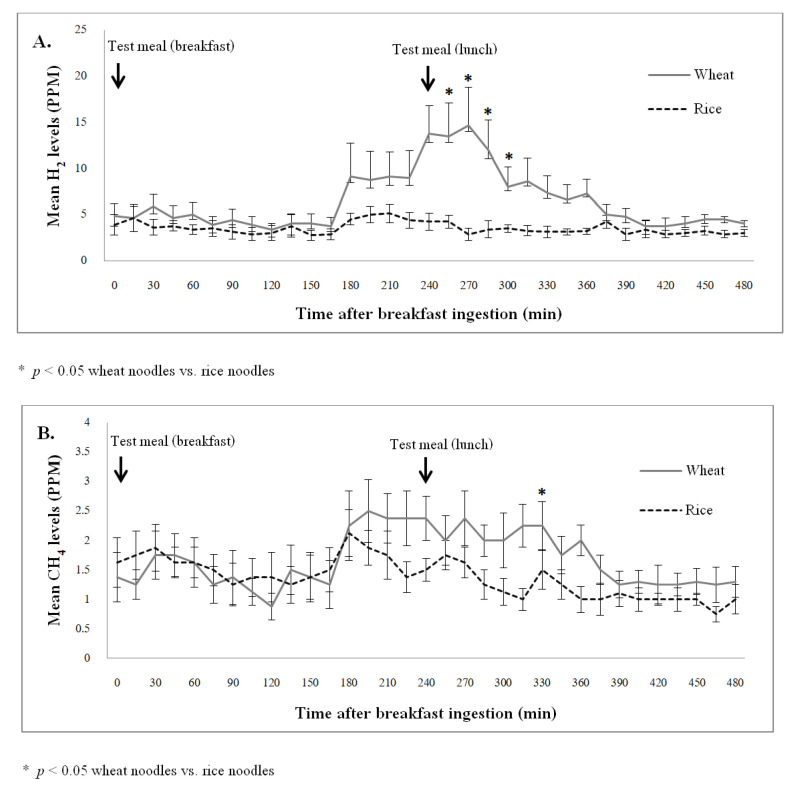
Exhaled gas concentrations after ingestion of different test meals (hydrogen, figure (**A**); methane, figure (**B**)).

The mean exhaled H_2_ and CH_4_ concentrations before and after breakfast were similar between the wheat and rice study arm (*p* > 0.05). From 15 min after the lunch, the wheat noodles were significantly associated with a higher exhaled H_2_ concentration than the rice noodles (*p* < 0.05), and the H_2_ concentration remained significantly different at 30 min, 45 min, and 60 min after the lunch (*p* < 0.05). Overall, after wheat ingestion, the exhaled CH_4_ concentration was higher than the levels after rice ingestion, with a significantly difference found at 90 min after lunch (*p* < 0.05). The exhaled H_2_ and CH_4_ concentration profiles during the study were demonstrated in Figure 3A,B.

The area under the curve (AUC) of H_2_ and CH_4_ concentration over 8 h after wheat ingestion were significantly greater than the levels after rice ingestion (AUC: H_2_ = 2541.6 ± 1765.0 vs. 1294.7 ± 750.8 ppm-min; CH_4_ = 631.9 ± 353.0 vs. 534.4 ± 323.2 ppm-min, *p* < 0.05). During a 4-h period after breakfast, the AUC of H_2_ and CH_4_ concentration were similar when comparing wheat and rice ingestion (AUC: H_2_ = 1256.3 ± 805.5 vs. 869.1 ± 571.8 ppm-min; CH_4_ = 372.2 ± 283.4 vs. 368.5 ± 218.8 ppm-min, *p* > 0.05). However, during a 4-h period after lunch, the AUC of H_2_ and CH_4_ concentration in the wheat study arm were significantly higher than the rice study arm (AUC: H_2_ = 1285.3 ± 959.5 vs. 425.6 ± 179.0 ppm-min; CH_4_ = 259.7 ± 69.6 vs. 165.9 ± 104.4 ppm-min, *p* < 0.05).

**Figure 4:**
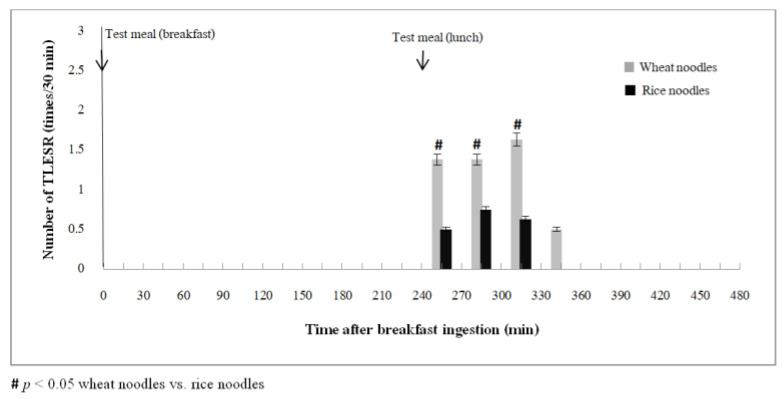
The number of TLESR per 30 min after lunch comparing between wheat and rice noodle ingestion. The area under the curve of exhaled H_2_ concentration 2 h after a wheat meal lunch (Figure 3A) significantly correlated with the number of TLESR events.

Figure 4 shows the number of TLESR 2 h after lunch. The area under the curve of exhaled H_2_ concentration 2 h after a wheat meal lunch significantly correlated with the number of TLESR events (r = 0.69, *p* = 0.04) and the regurgitation symptom severity score (r = 0.72, *p* = 0.03). Whereas the area under the curve of exhaled CH_4_ concentration 2 h after a wheat meal lunch did not correlate with the number of TLESR events and reflux symptoms severity (*p* > 0.05).

In the analysis of variance, there was no indication of a sequence effect, a period effect, or a carryover effect on the TLESR, GI symptoms, and gas production (*p* > 0.05).

## 4. Discussion

This study aims to evaluate the effects of high and low FODMAP meals in the form of wheat noodles and rice noodles, respectively, on transient lower esophageal sphincter relaxation (TLESR), intestinal gas production, postprandial heartburn, regurgitation, and other GI symptoms in overlapping GERD and non-constipation type IBS patients. We found that wheat noodles were significantly associated with more postprandial TLESR events than rice noodles. The difference in the rate of TLESR monitored by the HRiM catheter was observed immediately after lunchtime and continued for 90 min. Moreover, wheat noodles produced higher exhaled H_2_ and CH_4_ concentrations after lunch and higher postprandial regurgitation, bloating, satiety, and belching symptom severity scores than rice noodles. The finding suggests that due to an increase in the rate of TLESR after wheat noodles, high FODMAP meals were likely associated with intestinal gas production or colonic fermentation. 

The Monash University FODMAP diet application classified rice noodles as a low FODMAP food and wheat noodles as a high FODMAP food. Wheat-based food had been reported as the most problematic food on IBS patient symptoms. A serving size (cooked, 165 g) of wheat-based pasta contains 2.5 g of fructan, which is considerably high in FODMAP content [30]. In this study, we use 250 g rice noodles to represent a low FODMAP meal and 250 g wheat noodles to represent a high FODMAP meal. Previous studies in our center in patients with IBS showed that rice ingestion produces less intestinal gas, less bloating, and satiety scores than wheat [25,31]. This difference was not significant after breakfast but started immediately after lunchtime [25,31]. This finding suggests that rice noodles is completely absorbed in the small bowel, and wheat noodles, which is incompletely absorbed, was moved from the ileum into the large bowel, stimulated by lunch ingestion [32]. The other supporting data reported that bloating and abdominal distention in IBS patients was less in the morning but commonly higher at the end of the day [33]. Therefore, we decided to study the effects of high and low FODMAP meals on TLESR after lunch in this study.

The exhaled H_2_ concentrations after a wheat noodle lunch and the TLESR number are consistent with previous healthy-subject studies [21,23]. Intracolonic infusion of lactose and SCFAs, a result of carbohydrate fermentation, significantly decreased proximal gastric tone in a dose-dependent manner [21] and was associated with an increasing number of TLESR and TLESR associated with acid reflux episodes [23]. As a randomized crossover design, our study’s finding in GERD overlapping IBS patients suggests that FODMAP meals are likely to modulate TLESRs by colonic fermentation or intestinal H_2_ production. 

This study suggests the possible role of a low FODMAP diet as a non-pharmacologic treatment for GERD with overlapping IBS patients. We found that the severity of postprandial GER symptoms, bloating, satiety, and belching symptoms after rice noodles were significantly lower than after wheat noodle meals. A recent open-label study showed different findings [34]. They reported a comparable benefit of a 4-week low FODMAP diet and usual dietary advice (low-fat diet and avoid alcohol, caffeine, and overeating) on typical reflux symptoms and dyspepsia in patients who had PPI-refractory GERD. Associated IBS symptoms improved significantly only in the low FODMAP diet group. The 24-h pH-impedance monitoring parameters, in terms of total reflux events number, total acid exposure, total bolus exposure, nor a rate of patients with a positive symptomatic association, were not significantly different after 4 weeks of diet. However, most of the participants in this study had functional esophageal disorders, and the median (interquartile range) total acid exposure was only 1.1% (0.2–2.6). Therefore, this study could not completely ignore the beneficial effect of low FODMAPs in GERD patients with abnormal acid exposure time or gastroesophageal reflux number and non-PPI refractory GERD. Future studies on GERD patients who have esophagitis or significant acid exposure may provide different results. Geysen et al. recently reported that acute administration of a high caloric meal enriched with high fructan (40 g) in healthy volunteers did not induce GI symptoms, TLESR numbers, or reflux events during a five hour post-prandial period [35]. This finding may be due to a single meal administration, and the meal had not reached the colon where the fructan was fermented. Other factors may also have contributed to the different results on patient symptoms between studies. A study in twelve healthy subjects comparing a whole wheat bread and a rice pudding meal with equal calories showed that the wheat bread meal had longer gastric half-emptying times compared with the rice meal without significant differences in post-prandial fullness, bloating, distension, and nausea between meals [36]. Patients with GERD and healthy humans have been reported to have gastric accommodation responses to chili differently [37]. Thus, response to FODMAP diet in GERD patients and healthy volunteers may not be the same. 

Limitations of our study are: (1) it is not possible to exclude the effect of gluten on intestinal gas production and TLESR in this study as gluten is a component of wheat. Previous studies in healthy volunteers showed that gluten-containing wheat meals produce a significantly higher cumulative breath H_2_ excretion than after gluten-free wheat meals and gluten-free wheat meals with added gluten [38,39]. It is possible that the procedures for gluten extraction from the flour altered the carbohydrate, making it more absorbable. The effect of a gluten-free diet on preventing long-term recurrence of GERD symptoms after acid suppression withdrawal in adult celiac patients with nonerosive reflux disease has been reported [40], but the low FODMAP effect could not be excluded in this study. To our knowledge, there were no studies on the effect of gluten on the TLESR; (2) Wheat noodles and rice noodles are common foods in our country. Patients might have had different experiences with wheat noodles and rice noodles and expected different results. We could not make two different study meals identical looking and the amount of carbohydrate, protein, fat per gram, and dry weight in wheat and rice noodles are slightly different. However, we tried to minimize these limitations by avoiding informing the patients about the carbohydrate sources, FODMAPs, and expecting effects. Moreover, we used a crossover method with a one-week washout period, and we demonstrated the impact not only on the patient symptoms but on objective parameters, which are TLESR number and breath H_2_, and by blinding the study meals to the assessors; (3) A dinner meal before the study date may have affected baseline symptoms and baseline intestinal gas production. However, we instructed what patients should ingest and asked the patients to record their diet on the day before the study. This limitation seems to be minimal in our study as the baseline and morning H_2_ and CH_4_ gas productions were similar between the two test meals; (4) the sample size of eight was relatively small to demonstrate the difference of the severity of other GI symptoms, especially heartburn; (5) We did not perform an esophageal pH study in all patients. Although all the participants had no esophagitis, the study results could not be generalized to all GERD phenotypes, GERD patients without IBS, or GERD patients with constipation type IBS. Future studies on GERD patients with esophagitis, significant esophageal acid exposure, or GERD patients with constipation type IBS may provide different results. We excluded patients with constipation because it has been reported that intestinal gas production detected by exhaled gas can be increased in patients with hard stool (BSFS 1 or 2) [41], and constipation has been reported to be associated with GERD [42,43]; (6) Although the benefit of two meals (breakfast and lunch) from a low FODMAP diet was demonstrated in this study, an effect size is small and a long-term study has to be evaluated before recommending a low FODMAP diet in overlapping GERD-IBS to treat GERD and upper GI symptoms.

## 5. Conclusions

This study demonstrated that wheat noodles, a high FODMAP meal, produce more TLESRs, typical GERD—bloating, satiety, and belching—symptom scores after lunch compared to rice noodles, a low FODMAP meal. The effect of a high FODMAP meals on TLESRs is associated with an increase in intestinal gas production, confirming the role of colonic fermentation or colonic SCFA on the modulation of TLESRs. This study provides insight into the role of FODMAP dietary modification for treating patients with overlapping GERD and non-constipation type IBS.

## Figures and Tables

**Table 1:** Baseline gastrointestinal symptom scores on the study day.

	Wheat Noodles (*n* = 8)	Rice Noodles (*n* = 8)
GERD symptoms (Visual analog scale 0–10)		
Heartburn	1.0 (1.0–1.8)	1.0 (1.0–1.0)
Regurgitation	2.0 (1.0–3.0)	2.0 (2.0–2.8)
Other GI symptoms (Visual analog scale 0–10)		
Bloating	3.5 (2.3–4.0)	2.5 (1.3–3.0)
Satiety	3.0 (2.0–3.8)	2.0 (0.3–4.0)
Abdominal pain	1.5 (1.0–2.0)	1.0 (1.0–2.0)
Stool urgency	1.0 (1.0–1.0)	1.5 (1.0–2.0)
Nausea/Vomiting	2.0 (2.0–3.8)	2.0 (2.0–3.0)
Belching	2.0 (1.3–3.8)	2.0 (2.0–2.0)
Chest discomfort	0.00	0.00
Flatulence	1.0 (1.0–1.8)	1.0 (1.0–1.0)
Overall GI symptoms (Visual analog scale 0–10)	6.0 (5.3–7.0)	5.0 (4.3–6.0)
GERD, Gastroesophageal reflux disease; GI, Gastrointestinal		

Data expressed as median (interquartile ranges), *p* > 0.05, wheat noodles vs. rice noodle for all symptoms.

**Table 2:** Gastrointestinal symptom scores after lunch comparing wheat and rice noodle ingestion.

	Wheat Noodles (*n* = 8)	Rice Noodles (*n* = 8)
Overall GI symptoms (Visual analog scale 0–10) *	7.0 (6.0–8.8)	4.5 (3.3–6.0)
Average postprandial GI symptom scores (Visual analog scale 0–10)		
Heartburn	2.0 (0.1–3.6)	0.3 (0.0–1.6)
Regurgitation *	1.5 (0.0–6.1)	0.3 (0.0–0.9)
Bloating *	7.3 (6.1–7.9)	3.0 (0.3–5.8)
Satiety *	7.5 (6.6–8.9)	3.5 (1.1–6.1)
Abdominal pain	0.8 (0.0–2.1)	0.3 (0.0–1.6)
Stool urgency	0.3 (0.0–1.6)	0.0 (0.0–0.5)
Nausea/Vomiting	0.5 (0.1–1.9)	0.3 (0.0–0.9)
Belching *	3.5 (0.3–6.9)	0.5 (0.1–0.9)
Chest discomfort	0.3 (0.0–1.0)	0.3 (0.0–0.5)
Flatulence	1.0 (0.6–2.1)	1.3 (0.5–1.5)

Data expressed as median (Interquartile ranges), * *p* < 0.05, wheat noodles vs. rice noodles.

## Supplementary Materials

The following are available online at https://www.mdpi.com/article/10.3390/nu14091755/s1, Figure S1: The example of esophageal manometry tracing of a patient.

## References

[1] Lovell R.M., Ford A. (2012). Prevalence of gastro-esophageal reflux-type symptoms in individuals with irritable bowel syndrome in the community: A meta-analysis. Am. J. Gastroenterol..

[2] Nastaskin I., Mehdikhani E., Conklin J., Park S., Pimentel M. (2006). Studying the overlap between IBS and GERD: A systematic review of the literature. Dig. Dis. Sci..

[3] Mönnikes H., Heading R.C., Schmitt H., Doerfler H. (2011). Influence of irritable bowel syndrome on treatment outcome in gastroesophageal reflux disease. World J. Gastroenterol..

[4] McIntosh K., Reed D.E., Schneider T., Dang F., Keshteli A.H., De Palma G., Madsen K., Bercik P., Vanner S. (2017). FODMAPs alter symptoms and the metabolome of patients with IBS: A randomised controlled trial. Gut.

[5] Patcharatrakul T., Juntrapirat A., Lakananurak N., Gonlachanvit S. (2019). Effect of Structural Individual Low-FODMAP Dietary Advice vs. Brief Advice on a Commonly Recommended Diet on IBS Symptoms and Intestinal Gas Production. Nutrients.

[6] Wilson B., Rossi M., Kanno T., Parkes G.C., Anderson S., Mason A.J., Irving P.M., Lomer M.C., Whelan K. (2020). β-Galactooligosaccharide in Conjunction with Low FODMAP Diet Improves Irritable Bowel Syndrome Symptoms but Reduces Fecal Bifidobacteria. Am. J. Gastroenterol..

[7] Böhn L., Störsrud S., Liljebo T., Collin L., Lindfors P., Törnblom H., Simrén M. (2015). Diet low in FODMAPs reduces symptoms of irritable bowel syndrome as well as traditional dietary advice: A randomized controlled trial. Gastroenterology.

[8] Eswaran S.L., Chey W.D., Han-Markey T., Ball S., Jackson K. (2016). A Randomized Controlled Trial Comparing the Low FODMAP Diet vs. Modified NICE Guidelines in US Adults with IBS-D. Am. J. Gastroenterol..

[9] Goyal O., Batta S., Nohria S., Kishore H., Goyal P., Sehgal R., Sood A. (2021). Low fermentable oligosaccharide, disaccharide, monosaccharide, and polyol diet in patients with diarrhea-predominant irritable bowel syndrome: A prospective, randomized trial. J. Gastroenterol. Hepatol..

[10] Halmos E.P., Power V.A., Shepherd S.J., Gibson P.R., Muir J.G. (2014). A diet low in fodmaps reduces symptoms of irritable bowel syndrome. Gastroenterology.

[11] Harvie R.M., Chisholm A.W., Bisanz J.E., Burton J.P., Herbison P., Schultz K., Schultz M. (2017). Long-term irritable bowel syndrome symptom control with reintroduction of selected FODMAPs. World J. Gastroenterol..

[12] Pedersen N., Ankersen D.V., Felding M., Wachmann H., Végh Z., Molzen L., Burisch J., Andersen J.R., Munkholm P. (2017). Low-FODMAP diet reduces irritable bowel symptoms in patients with inflammatory bowel disease. World J. Gastroenterol..

[13] Staudacher H.M., Lomer M.C., Anderson J.L., Barrett J.S., Muir J.G., Irving P.M., Whelan K. (2012). Fermentable carbohydrate restriction reduces luminal bifidobacteria and gastrointestinal symptoms in patients with irritable bowel syndrome. J. Nutr..

[14] Staudacher H.M., Lomer M.C.E., Farquharson F.M., Louis P., Fava F., Franciosi E., Scholz M., Tuohy K.M., Lindsay J.O., Irving P.M. (2017). A Diet Low in FODMAPs Reduces Symptoms in Patients with Irritable Bowel Syndrome and A Probiotic Restores Bifidobacterium Species: A Randomized Controlled Trial. Gastroenterology.

[15] Zahedi M.J., Behrouz V., Azimi M. (2018). Low fermentable oligo-di-mono-saccharides and polyols diet versus general dietary advice in patients with diarrhea-predominant irritable bowel syndrome: A randomized controlled trial. J. Gastroenterol. Hepatol..

[16] Zhang Y., Feng L., Wang X., Fox M., Luo L., Du L., Chen B., Chen X., He H., Zhu S. (2021). Low fermentable oligosaccharides, disaccharides, monosaccharides, and polyols diet compared with traditional dietary advice for diarrhea-predominant irritable bowel syndrome: A parallel-group, randomized controlled trial with analysis of clinical and microbiological factors associated with patient outcomes. Am. J. Clin. Nutr..

[17] Katz P.O., Gerson L.B., Vela M.F. (2013). Guidelines for the diagnosis and management of gastroesophageal reflux disease. Am. J. Gastroenterol..

[18] Ness-Jensen E., Hveem K., El-Serag H., Lagergren J. (2015). Lifestyle Intervention in Gastroesophageal Reflux Disease. Clin. Gastroenterol. Hepatol..

[19] Fox M., Barr C., Nolan S., Lomer M., Anggiansah A., Wong T. (2007). The effects of dietary fat and calorie density on esophageal acid exposure and reflux symptoms. Clin. Gastroenterol. Hepatol. Off. Clin. Pract. J. Am. Gastroenterol. Assoc..

[20] Sethi S., Richter J.E. (2017). Diet and gastroesophageal reflux disease: Role in pathogenesis and management. Curr. Opin. Gastroenterol..

[21] Ropert A., Cherbut C., Roze C., Le Quellec A., Holst J.J., Fu-Cheng X., Varannes S.B.D., Galmiche J.P. (1996). Colonic fermentation and proximal gastric tone in humans. Gastroenterology.

[22] Zerbib F., Varannes S.B.D., Rozé D.C., Galmiche J.P. (1996). Simultaneous study of tones of the lower esophageal sphincter and proximal stomach in healthy humans. Gastroentérologie Clin. Biol..

[23] Piche T., Zerbib F., Varannes S.B.D., Cherbut C., Anini Y., Roze C., Le Quellec A., Galmiche J.-P. (2000). Modulation by colonic fermentation of LES function in humans. Am. J. Physiol. Liver Physiol..

[24] Zerbib F., Varannes S.B.D., Scarpignato C., Leray V., D’Amato M., Rozé C., Galmiche J.-P. (1998). Endogenous cholecystokinin in postprandial lower esophageal sphincter function and fundic tone in humans. Am. J. Physiol. Content.

[25] Linlawan S., Patcharatrakul T., Gonlachanvit S. (2019). Effect of Rice, Wheat and Mung Bean Ingestion on Intestinal Gas Production and Postprandial Gastrointestinal Symptoms in Non-constipation Irritable Bowel Syndrome Patients. Nutrients.

[26] USDA Food Data Central. https://ndb.nal.usda.gov/ndb/search/list.

[27] Simren M., Stotzer P.O. (2006). Use and abuse of hydrogen breath tests. Gut.

[28] Roman S., Zerbib F., Belhocine K., Varannes S.B.D., Mion F. (2011). High resolution manometry to detect transient lower oesophageal sphincter relaxations: Diagnostic accuracy compared with perfused-sleeve manometry, and the definition of new detection criteria. Aliment. Pharmacol. Ther..

[29] Piche T., Varannes S.B.D., Sacher-Huvelin S., Holst J.J., Cuber J.C., Galmiche J.P. (2003). Colonic fermentation influences lower esophageal sphincter function in gastroesophageal reflux disease. Gastroenterology.

[30] Shepherd S.J., Gibson P.R. (2006). Fructose malabsorption and symptoms of irritable bowelsyndrome: Guidelines for effective dietary management. Am. Diet. Assoc..

[31] Patcharatrakul T., Linlawan S., Plaidum S., Gonlachanvit S. (2021). The Effect of Rice vs. Wheat Ingestion on Postprandial Gastroesophageal Reflux (GER) Symptoms in Patients with Overlapping GERD-Irritable Bowel Syndrome (IBS). Foods.

[32] Gonlachanvit S. (2010). Are rice and spicy diet good for functional gastrointestinal disorders?. J. Neurogastroenterol. Motil..

[33] Houghton L.A., Lea R., Agrawal A., Reilly B., Whorwell P. (2006). Relationship of abdominal bloating to distention in irritable bowel syndrome and effect of bowel habit. Gastroenterology.

[34] Rivière P., Vauquelin B., Rolland E., Melchior C., Roman S., Varannes S.B.D., Mion F., Gourcerol G., Sacher-Huvelin S., Zerbib F. (2021). Low FODMAPs diet or usual dietary advice for the treatment of refractory gastroesophageal reflux disease: An open-labeled randomized trial. Neurogastroenterol. Motil..

[35] Geysen H., Gielis E., Deloose E., Vanuytsel T., Tack J., Biesiekierski J.R., Pauwels A. (2019). Acute administration of fructans increases the number of transient lower esophageal sphincter relaxations in healthy volunteers. Neurogastroenterol. Motil..

[36] Marciani L., Pritchard S.E., Hellier-Woods C., Costigan C., Hoad C., Gowland P., Spiller R.C. (2013). Delayed gastric emptying and reduced postprandial small bowel water content of equicaloric whole meal bread versus rice meals in healthy subjects: Novel MRI insights. Eur. J. Clin. Nutr..

[37] Patcharatrakul T., Kriengkirakul C., Chaiwattanarat T., Gonlachanvit S. (2020). Acute Effects of Red Chili, a Natural Capsaicin Receptor Agonist, on Gastric Accommodation and Upper Gastrointestinal Symptoms in Healthy Volunteers and Gastroesophageal Reflux Disease Patients. Nutrients.

[38] Di Stefano M., Maffè G.C., Bergonzi M., Mengoli C., Formagnana P., Di Sabatino A., Corazza G.R. (2015). The effect of gluten on intestinal fermentation, gastric and gallbladder emptying in healthy volunteers. Dig. Liver Dis. Off. J. Ital. Soc. Gastroenterol. Ital. Assoc. Study Liver.

[39] Anderson I.H., Levine A.S., Levitt M.D. (1981). Incomplete absorption of the carbohydrate in all-purpose wheat flour. N. Engl. J. Med..

[40] Usai P., Manca R., Cuomo R., Lai M.A., Russo L., Boi M.F. (2008). Effect of gluten-free diet on preventing recurrence of gastroesophageal reflux disease-related symptoms in adult celiac patients with nonerosive reflux disease. J. Gastroenterol. Hepatol..

[41] Di Stefano M., Mengoli C., Bergonzi M., Miceli E., Pagani E., Corazza G.R. (2014). Hydrogen breath test in patients with severe constipation: The interference of the mixing of intestinal content. Neurogastroenterol. Motil..

[42] Baran M., Cagan Appak Y., Karakoyun M., Yalcinkaya S., Eliacik K., Dundar B.N. (2017). The overlap of gastroesophageal reflux disease and functional constipation in children: The efficacy of constipation treatment. Eur. J. Gastroenterol. Hepatol..

[43] Hosseini M., Salari R., Rad M.A., Salehi M., Birjandi B., Salari M. (2018). Comparing the Effect of Psyllium Seed on Gastroesophageal Reflux Disease with Oral Omeprazole in Patients With Functional Constipation. J. Evid.-Based Integr. Med..

