# Supplementary figures and images for: The Effect of Fermentable, Oligosaccharides, Disaccharides, Monosaccharides, and Polyols (FODMAP) Meals on Transient Lower Esophageal Relaxations (TLESR) in Gastroesophageal Reflux Disease (GERD) Patients with Overlapping Irritable Bowel Syndrome (IBS)
Source: Nutrients. 2022 Apr 22;14(9):1755. doi: 10.3390/nu14091755 (PMC9101233; doi:10.3390/nu14091755)

**Figure S1.** The example of esophageal manometry tracing of a patient.

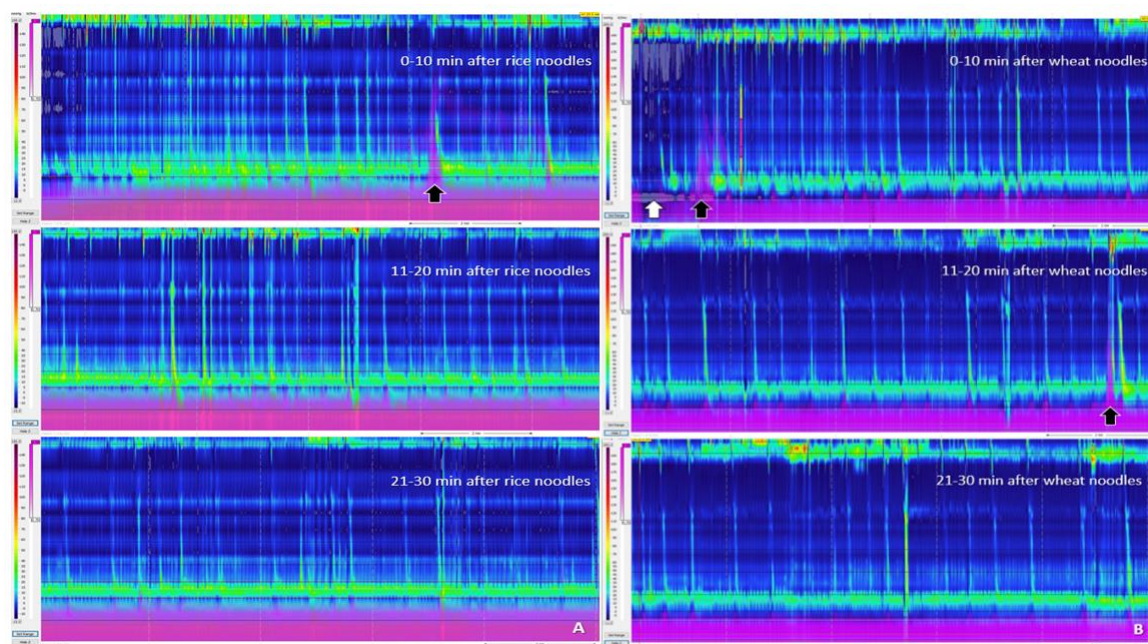

Supplement: Supplementary file 1 [file nutrients-14-01755-s001.zip › nutrients-1602772-supplementary.pdf]
